# Maintenance of adaptive differentiation by *Wolbachia *induced bidirectional cytoplasmic incompatibility: the importance of sib-mating and genetic systems

**DOI:** 10.1186/1471-2148-9-185

**Published:** 2009-08-04

**Authors:** Antoine Branca, Fabrice Vavre, Jean-François Silvain, Stéphane Dupas

**Affiliations:** 1 Unité de Recherche IRD 072, CNRS UPR9034, Laboratoire Evolution, Génome et Spéciation, Gif-sur-Yvette, France; 2 Université Paris-Sud 11, Orsay, France; 3 CNRS UMR5558, Laboratoire de Biométrie et Biologie Evolutive, Villeurbanne, France; 4 Université Lyon 1, Villeurbanne, France; 5 Unité de Recherche IRD 072, Pontificia Universidad Católica del Ecuador, Quito, Ecuador

## Abstract

**Background:**

Bacteria of the genus *Wolbachia *are reproductive parasites widespread among arthropods. The most common effect arising from the presence of *Wolbachia *in a population is Cytoplasmic Incompatibility (CI), whereby postmating reproductive isolation occurs in crosses between an infected male and an uninfected female, or when a male is infected with a different strain of *Wolbachia *to that of the female (bidirectional CI). Previous theoretical models have demonstrated that bidirectional CI can contribute to the genetic divergence of populations in haploid and diploid organisms. However, haplodiploid organisms were not considered in these models even though they include *Nasonia *parasitoid wasps – the best example of the implication of *Wolbachia *in ongoing speciation. Moreover, previous work did not investigate inbreeding mating systems, which are frequently observed in arthropod species.

**Results:**

We developed a stochastic two-island model which simulated three genetic scenarios, diploidy, haploidy, and haplodiploidy, with two CI phenotypes being considered for the latter: (1) male development of female progeny; and (2) mortality of fertilized eggs. We also investigated the effect of varying the proportion of sib mating. In the model each allopatric population was initially fixed for a single allele at a nuclear locus under positive selection and infected with one strain of *Wolbachia*. Each simulation presupposed that the two populations were fixed for a different allele and a different strain of *Wolbachia*. The degree of genetic differentiation observed in the locus under selection due to bidirectional CI was much lower for the two haplodiploid phenotypes than for either diploids or haploids. Furthermore, we demonstrated that sib-mating may compensate for the lower efficiency of bidirectional CI in haplodiploids by maintaining genetic divergence.

**Conclusion:**

Our model suggests that maintenance of genetic differentiation facilitated by *Wolbachia *is more likely to occur in diploids and haploids than in haplodiploids. However, increasing the level of sib-mating may compensate for the weak effect of bidirectional CI in haplodiploids. Our work therefore gives a potential explanation for why the haplodiploid *Nasonia *species, which are infected with bidirectionally incompatible *Wolbachia *strains and undergo sib-mating, have differentiated genetically and maintained this differentiation without premating isolation.

## Background

Insects are frequently infected with bacterial symbionts. Many of them such as *Wolbachia*, *Cardinium *or *Spiroplasma *manipulate the reproduction of their hosts for their own advantage using different effects such as: feminization of genetic males, increase of male mortality (male killing), thelytokous parthenogenesis induction (PI) and, most commonly, cytoplasmic incompatibility (CI) [1-9]. All of these reproductive parasites are transferred vertically from mother to progeny via the cytoplasm of the eggs.

Unidirectional CI may occur when an uninfected female mates with an infected male [2,10]. The infected male's sperm is modified by the bacterial infection and, as a result, cannot fertilize eggs from female unless they are rescued by the same strain being present in the cytoplasm of the eggs [11,12]. In diploids, expression of CI results in no offspring development. In haplodiploids, the two CI phenotypes *female egg mortality *(FM) and *male development of female eggs *(MD), result in a male biased sex-ratio in the offspring [13-15]. In the FM phenotype, a reduction in the number of progeny is observed because only unfertilized eggs can develop into males (fertilized eggs being developmentally lethal) [13,14]. In the MD phenotype, the number of progeny is not affected in incompatible matings as both fertilized and unfertilized eggs develop into males due to the complete haploidization of fertilized eggs [15]. In diploid and haplodiploid genetic systems, the symbiont increases in frequency in the insect population by allowing infected females to produce more daughters than uninfected females increasing the reproductive success of infected females [14,16].

In addition to unidirectional CI, bidirectional CI may occur when the parents are infected by different strains of bacteria [15]. By generating such bidirectional reproductive incompatibilities, it has been suggested that CI promotes speciation [2,17-20]. *Wolbachia *is the most common of the endosymbionts causing CI, and has recently been estimated to infect approximately 66% of all insect species, with prevalence rates within species ranging between 10–90% [21]. The clearest example of *Wolbachia *inducing diversification is described in the three parasitic wasps of *Nasonia *species. In this haplodiploid insect, there is no premating isolation, but postmating isolation occurs as a result of bidirectional CI [22,23]. It is therefore suggested that *Wolbachia *bacteria are the main agent preventing mixing between *Nasonia *species. However a direct role of *Wolbachia *in speciation remains a controversial topic [24,25]. For example, a study of isofemale lines from natural populations of *Drosophila simulans *infected with different bidirectionally incompatible *Wolbachia *strains, showed no association between *Wolbachia *strain and genetic divergence at neutral nuclear markers, and no evidence of assortative mating behavior [26]. This case suggests that CI alone cannot induce speciation in *Drosophila*. In contrast, another study of two *Drosophila *species showed that unidirectional CI leads to behavioral recognition and subsequent avoidance of the *Wolbachia *infected species by the uninfected one, hence maintaining premating isolation between species [27].

In an effort to investigate the possibility of CI promoting speciation, several theoretical models have been developed which focus on the impact of CI on genetic divergence. For example, theoretical frameworks showed that CI may strengthen genetic divergence between populations [28,29] and, moreover, in some situations, bidirectional CI selects for premating isolation and could lead to speciation [30]. However, previous models on the effect of CI on genetic differentiation [28-31] did not consider some important points. (*i*) The genetic system of haplodiploid parasitoids differs markedly to that of haploids and diploids, yet the clearest evidence of the *Wolbachia *role in early speciation events is cited as being in the haplodiploid *Nasonia *species complex [22]. (*ii*) The MD and FM phenotypes common to haplodiploids may have different effects on genetic differentiation [14,32]. (*iii*) In parasitoids, selection acts preferentially on females because ecological niches are generally defined by the female choice of host [33], and by the female injecting into the host many of the factors required for development, including venom, ovarian proteins [34] and polydnaviruses [35]. (*iv*) Inbreeding may play a role in enhancing genetic differentiation among populations; near complete outbreeding, sib-mating, and parthenogenesis can all be observed within haplodiploid parasitoid species [36-38]. Furthermore, it has been previously demonstrated that sib-mating modifies the invasion dynamics of the *Wolbachia *infection [39,40] Finally, (v) Using a stochastic approach allows a finite population to be defined and enables the incorporation of genetic drift and other random evolutionary effects. In particular, *Wolbachia *spread dynamics are represented differently under a stochastic modeling simulation [41].

**Figure 1:**
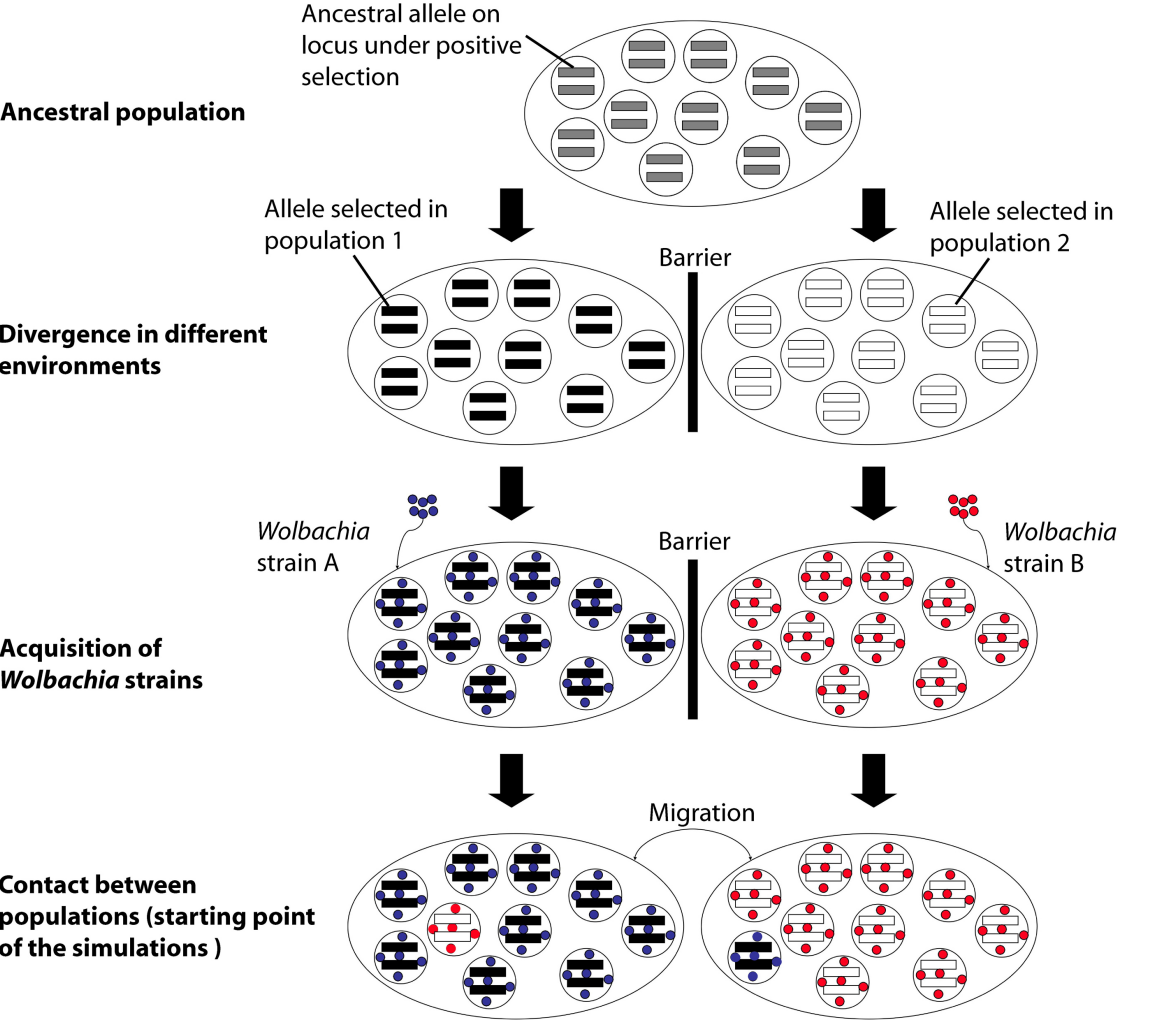
**Model scenario**. This figure represents a schematic view of the evolutionary scenario implemented in the model for diploids. An ancestral population is first separated into two populations. Each population is adapted to its environment at a single nuclear locus which is under positive selection. After different strains of *Wolbachia *had invaded the two populations and reached saturation point, migration was permitted between the populations. Simulations started at this step.

In this paper, we used a model similar to that of Telschow *et al*. [28] to investigate how CI contributes to maintaining divergence between two populations which have undergone different selection regimes, but after which nuclear genes and *Wolbachia *can be exchanged (Figure 1). Our approach is the first to (*i*) integrate different levels of ploidy, (*ii*) integrate different CI phenotypes, (*iii*) consider female only selection, (*iv*) allow sib-mating, and (v) use a stochastic model.

## Methods

### The model

As in previous models [28,30], simulations started after the contact of two populations, each infected with a different *Wolbachia *strain (Figure 1). Each population is monitored at a single nuclear locus under positive selection in females only, and each population is fixed for a different allele at this locus. We assumed a finite and constant population size *N *for each generation *g*. Each individual, male or female, has a probability *m *of migrating to the other population. Parameter *s*_*i *_is the selective coefficient, which is applied to a female possessing an allele not locally adapted to population *i *on the selected nuclear locus.

The two populations each harbored a different *Wolbachia *strain, either *A *or *B*, which were bidirectionally incompatible. We considered that infection by *Wolbachia *had no fitness effect. Each individual of the next generation had a probability *t *of receiving *Wolbachia *from its mother. The probability of either bacterial strain *A *or *B *to express CI was *I*_*A *_and *I*_*B *_respectively.

Following Engelstädter *et al*. and Branca and Dupas [39,40], we used a probability *χ *of each female mating with a brother. The proportion of female offspring, *P*_*SR*_, was fixed to 1 male for 3 females, (e.g. for gregarious parasitoids [42]). Indeed, evolutionary stable sex-ratios are predicted to be female-biased when there is local mate competition [43]. Note however, that we did not see any significant effects of the primary sex ratio on either maintenance of *Wolbachia *diversity or the allelic frequency dynamics of the nuclear locus (results not shown).

Our model called *CIParasitoid *was developed using the "R" language and software R 2.8.0 [44]. The package includes helps and can be installed using standard R commands (see Additional files 1, 2, 3 and 4).

### Selection modeling

In the two populations, females were fixed for a different allele on a single selected nuclear locus *V*. Typically this situation could represent the specialization of a parasitoid wasp (such as *Nasonia*) to different insect hosts. Thus, the locus *V *governed the capacity to successfully parasitize a host. This locus bore two alleles *V*_1 _specialized on host 1 and *V*_2 _specialized on host 2. We assumed semi-dominance of the alleles on locus *V *meaning that heterozygotes randomly expressed one of the two alleles. Consequently, in the haplodiploid genetic system, for i≠j the probabilities of successful development of the progeny in the host *i *were 1,  and 1-*s*_*i *_for the homozygotes *V*_i_*V*_i_, heterozygotes *V*_i_*V*_j _and homozygotes *V*_*j*_*V*_*j *_respectively. In the haploid case, these probabilities were 1 and 1-*s*_*i *_for the genotypes *V*_i _and *V*_*j *_respectively. In the algorithm, the reproductive females were therefore sampled according to their genotypes on the locus *V *while each male had the same probability of being sample, independently of its genotype.

As a result, we can calculate *F'*, the expected number of reproductive females of each genotype after the selection process in haplodiploids and diploids for population *i*, as follows:



And in haploids, as:



After this selection process, the program recorded all the reproductive individuals.

### Cytoplasmic Incompatibility modeling

An incompatible cross occurred when an infected male mated with a female which was either uninfected or infected with a different bacterial strain.

In diploids and haploids, when CI occurred no offspring were produced. For haplodiploids, the CI effect was assumed to either result in all MD or all FM phenotypes. Consequently, for the MD phenotype, CI resulted in the development of only male progeny; for the FM phenotype, female progeny resulting from incompatible crosses did not developed and the number of progeny therefore decreased. To account for this, the probability of producing no offspring was equal to the primary sex ratio, *P*_*SR*_, which was used as the probability of producing a female.

### Estimating genetic divergence and *Wolbachia *diversity maintenance

Previous studies have measured genetic divergence induced by bidirectional CI on a selected locus by quantifying the reduction in the effective migration rate, or the production of hybrid offspring [28]. In the haplodiploid model, this variable is not easily estimated because CI does not result in the death of offspring but rather in the production of males. Therefore, this caused complications in the calculation of the reduction of the effective migration rate. Consequently, the effect of CI on genetic divergence was estimated by calculating a *Fst *on the selected nuclear locus as follows:



where *p*_*i *_and *q*_*i *_are the frequencies of allele 1 and 2 respectively in population *i*.

The maintenance of *Wolbachia *diversity, namely *W*, was measured by the probability of maintaining each *Wolbachia *strain in its respective population at a frequency above an arbitrary threshold of 75%.

### Default parameters of the simulations

By default, the population size was set to *N *= 50 individuals in each population, allowing for a high drift effect in the system, with simulations stopping after *g *= 100 generations. Nevertheless, higher population sizes and longer running time were tested. The default values of parameters were set to *m *= 0.5, *t *= 1, *s*_*i *_= 0.5 and *χ *= 0. In all simulations, we set *s*_1 _= *s*_2_. Four different situations were calculated for bidirectional CI ranging from no CI to high levels of CI: (*I*_*A *_= 0; *I*_*B*_= 0), (*I*_*A *_= 0.5; *I*_*B *_= 0.75), (*I*_*A *_= 0.75; *I*_*B *_= 0.75) and (*I*_*A *_= 1; *I*_*B *_= 0.75). To test the significance of bidirectional CI on genetic divergence, we used a non-parametric unilateral Mann-Whitney test on *Fst *values. The H_0 _tested was "*Fst *in the bidirectional CI case was equal to *Fst *in the case where *I*_*A *_= 0 and *I*_*B *_= 0". The H_1 _tested was "*Fst *in the bidirectional CI case was superior to *Fst *in the case where *I*_*A *_= 0 and *I*_*B *_= 0". This latter case was equivalent to a case without *Wolbachia *(i.e. *Wolbachia *therefore behaved as mitochondria). *Fst *and *W *were calculated after 1000 repetitions.

## Results

### Differentiation at the selected nuclear locus due to bidirectional CI in the different genetic systems (Table 1; Figure 2; Figure 3)

**Table 1:** Test of the impact of bidirectional CI on genetic divergence for different values of *m*

Migration rate	0.2	0.175	0.15	0.125	0.1	0.075	0.05	0.025	0
	Diplo	Diplo	Diplo	Diplo	Diplo	Diplo	Diplo	Diplo	Diplo
*I*_*A *_= 0.75		Haplo	Haplo	Haplo	Haplo	Haplo	Haplo	Haplo	Haplo
*I*_*B *_= 1			HDMD	HDFM	HDFM	HDFM	HDFM	HDFM	HDFM
				HDMD	HDMD	HDMD	HDMD	HDMD	HDMD
	Diplo	Diplo	Diplo	Diplo	Diplo	Diplo	Diplo	Diplo	Diplo
*I*_*A *_= 0.75		Haplo	Haplo	Haplo	Haplo	Haplo	Haplo	Haplo	Haplo
*I*_*B *_= 0.75			HDMD	HDFM	HDFM	HDFM	HDFM	HDFM	HDFM
				HDMD	HDMD	HDMD	HDMD	HDMD	HDMD

				Diplo	Diplo	Diplo	Diplo	Diplo	Diplo
*I*_*A *_= 0.5					Haplo	Haplo	Haplo	Haplo	Haplo
*I*_*B *_= 0.75					HDFM	HDFM	HDFM	HDFM	HDFM
						HDMD	HDMD	HDMD	HDMD

Each box is filled with the name of the system where bidirectional CI significantly increased the *Fst *value on the selected locus (Mann-Whitney test, *p*-value = 0.05). Diplo = Diploids, Haplo = Haploids, HDFM = Haplodiploid Female Mortality and HDMD = Haplodiploid Male Development. Fixed parameters were set to *s*_*i *_= 0.5, *P*_*SR *_= 0.75, *χ *= 0, *t *= 1, *g *= 100, *N *= 50.

**Figure 2:**
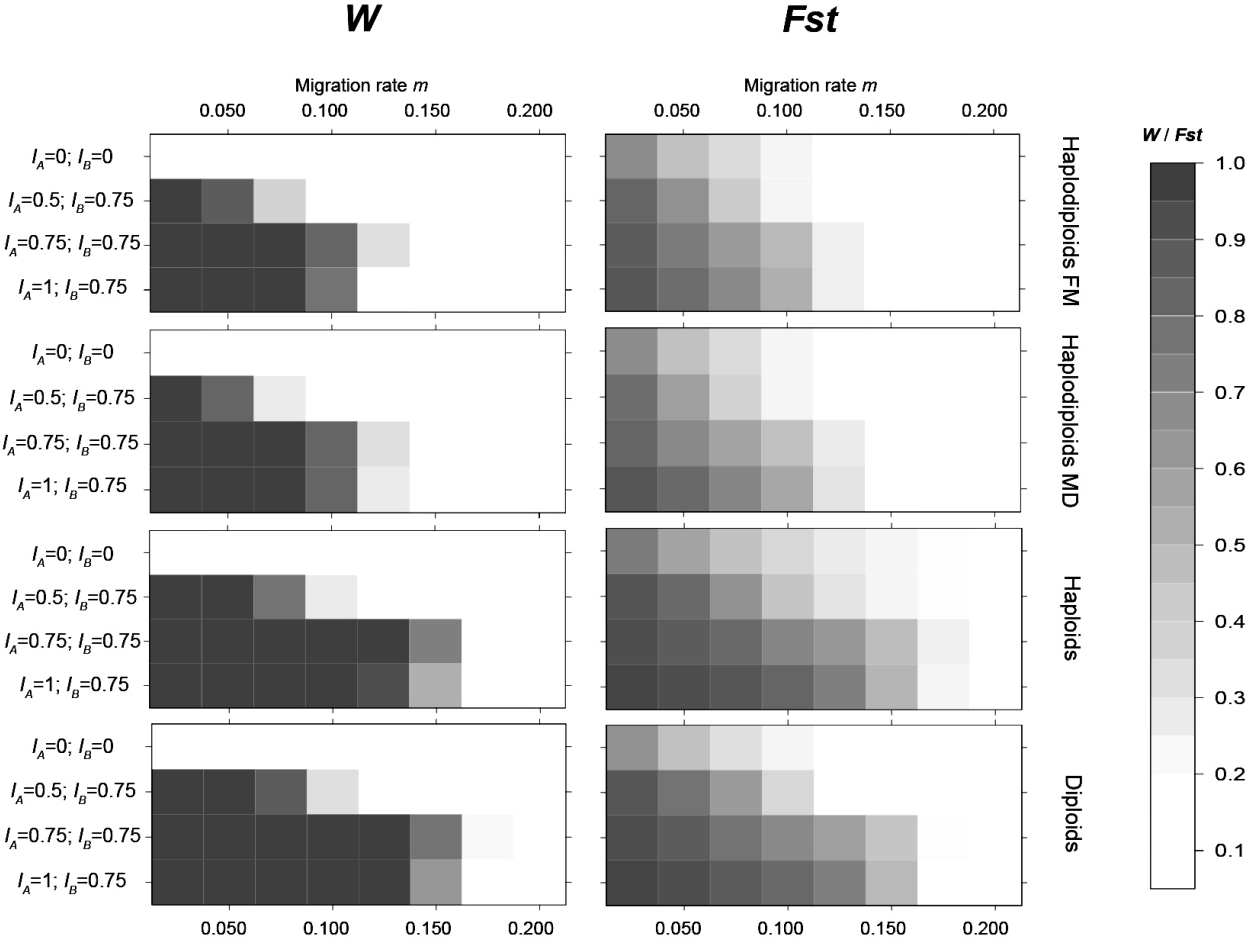
**Effect of migration rate *m *on idirectional CI and genetic divergence**. A representation of the evolution of *W *and *Fst *for different bidirectional CI situations (no CI (*I*_*A *_= *I*_*B *_= 0), (*I*_*A *_= 0.5; *I*_*B *_= 0.75), (*I*_*A *_= 0.75;*I*_*B *_= 0.75) (*I*_*A *_= 1;*I*_*B *_= 0.75)) and migration rate *m*. Other parameter values are *s*_*i *_= 0.5, *P*_*SR *_= 0.75, *χ *= 0, *t *= 1, *g *= 100, *N *= 50. Each graph represents each phenotype tested (Haplodiploids FM and MD, diploids and haploids). *W *is represented on the left and *Fst *on the right. The higher the value for each variable, the darker the box.

**Figure 3:**
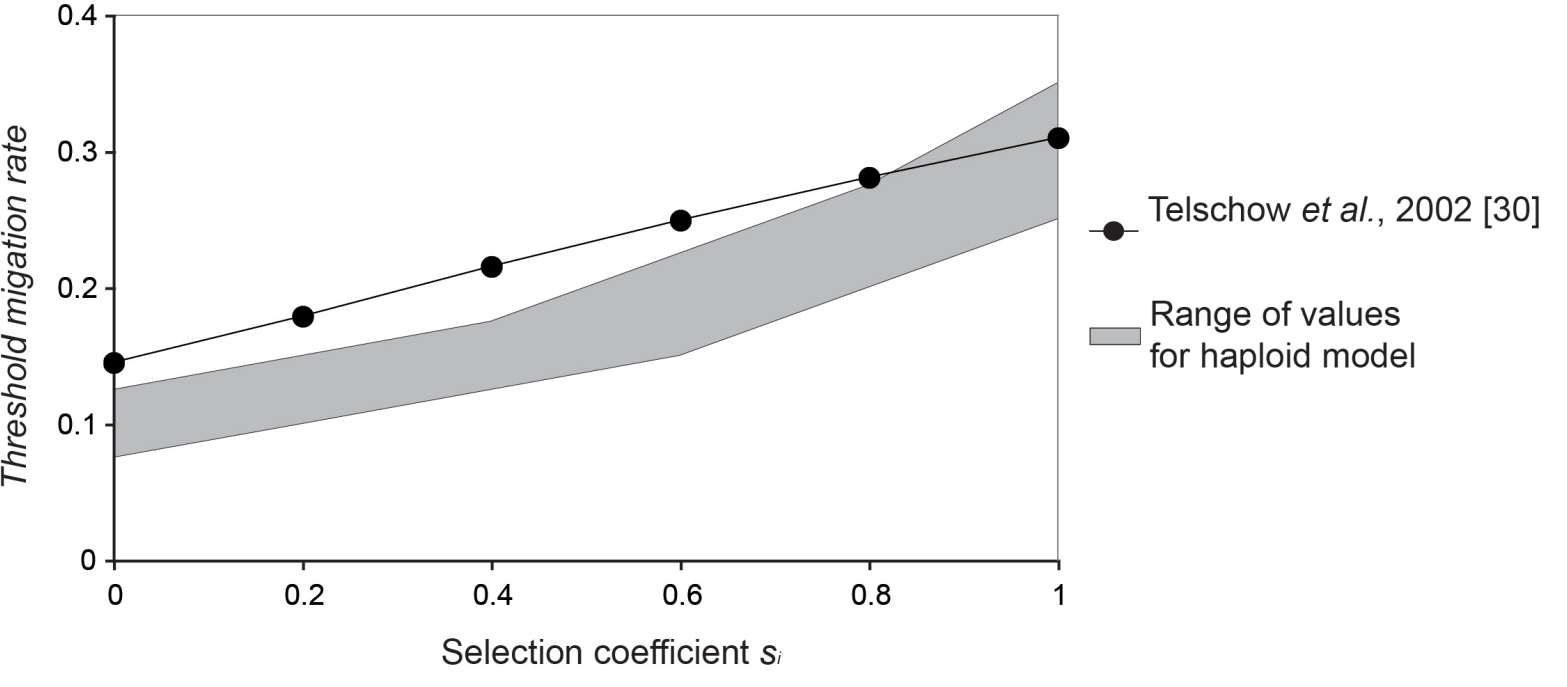
**Comparison of the threshold migration rate in stochastic vs deterministic models**. In the work by Telschow *et al*. [28], the threshold migration rate was defined as the migration rate beyond which *Wolbachia *diversity is no longer maintained. In our haploid model, a threshold migration range was represented as the migration rate valueswhere *W *drops from 0.95 to 0.05. The true threshold migration rate was assumed to be included in that range of migration rate values. Other parameter values were fixed at the same values as Telschow *et al*., 2002 [28]: *I*_*A *_= 0.8, *I*_*B *_= 0.8, *t *= 0.99, *sr *= 0.5.

Firstly, our results showed that *W *reached zero beyond a certain migration rate. It means that one *Wolbachia *strain has succeeded in saturating both populations (Figure 2). The previous model of Telschow *et al*. named this migration rate the *threshold migration rate *[28]. Threshold migration rates were lower in our stochastic model than in the previous deterministic model (Figure 3). However the threshold became closer at higher selection coefficients. Indeed, the threshold value was the same for both models where *s*_*i *_= 1.

Secondly, for all genetic systems, we observed that *Fst *reached significantly higher values when bidirectional CI occurred than in situations with selection only (Table 1 and Figure 2). Bidirectional CI is therefore a force that increased genetic divergence at a selected locus.

Thirdly, by comparing the different genetic systems, we observed that *threshold migration rates *for *W *were lower for haplodiploids than for diploids and haploids (e.g Figure 2a for *I*_*A *_= 1 and *I*_*B *_= 0.75, between 0.1 and 0.125 for haplodiploids and between 0.150 and 0.175 for haploids and diploids). In the same manner, lower *Fst *values were reached in the two haplodiploid phenotypes than in haploids and diploids. Indeed, in the three sets of incompatibility rates, *I*_*A *_and *I*_*B *_(Figure 2), *Fst *values in haplodiploids were never significantly higher than values in diploid and haploids (Mann-Whitney p < 0.001). In conclusion, when both *Wolbachia *strains were maintained, enhancement of genetic divergence by bidirectional CI was weaker in haplodiploids than in other genetic systems.

### Influence of selection on divergence under bidirectional CI (Table 2; Figure 4)

**Table 2:** Test of the impact of bidirectional CI on genetic divergence for different value of *s*_*i*_

Selection strength	0	0.125	0.25	0.375	0.5	0.625	0.75	0.875	1
	Diplo	Diplo	Diplo	Diplo	Diplo	Diplo	Diplo	Diplo	Diplo
*I*_*A *_= 0.75	Haplo	Haplo	Haplo	Haplo	Haplo	Haplo	Haplo	Haplo	Haplo
*I*_*B *_= 1			HDFM	HDFM	HDFM	HDFM	HDFM	HDFM	HDFM
				HDMD	HDMD	HDMD	HDMD	HDMD	HDMD
	Diplo	Diplo	Diplo	Diplo	Diplo	Diplo	Diplo	Diplo	Diplo
*I*_*A *_= 0.75	Haplo	Haplo	Haplo	Haplo	Haplo	Haplo	Haplo	Haplo	Haplo
*I*_*B *_= 0.75			HDFM	HDFM	HDFM	HDFM	HDFM	HDFM	HDFM
				HDMD	HDMD	HDMD	HDMD	HDMD	HDMD

				Diplo	Diplo	Diplo	Diplo	Diplo	Diplo
*I*_*A *_= 0.5					Haplo	Haplo	Haplo	Haplo	Haplo
*I*_*B *_= 0.75					HDFM	HDFM	HDFM	HDFM	HDFM
						HDMD	HDMD	HDMD	HDMD

Each box is filled with the name of the system where bidirectional CI significantly increased the *Fst *value on the selected locus (Mann-Whitney test, *p*-value = 0.05).

Diplo = Diploids, Haplo = Haploids, HDFM = Haplodiploid Female Mortality and HDMD = Haplodiploid Male Development. Fixed parameters were set to *m *= 0.1, *P*_*SR *_= 0.75, *χ *= 0, *t *= 1, *g *= 100, *N *= 50.

**Figure 4:**
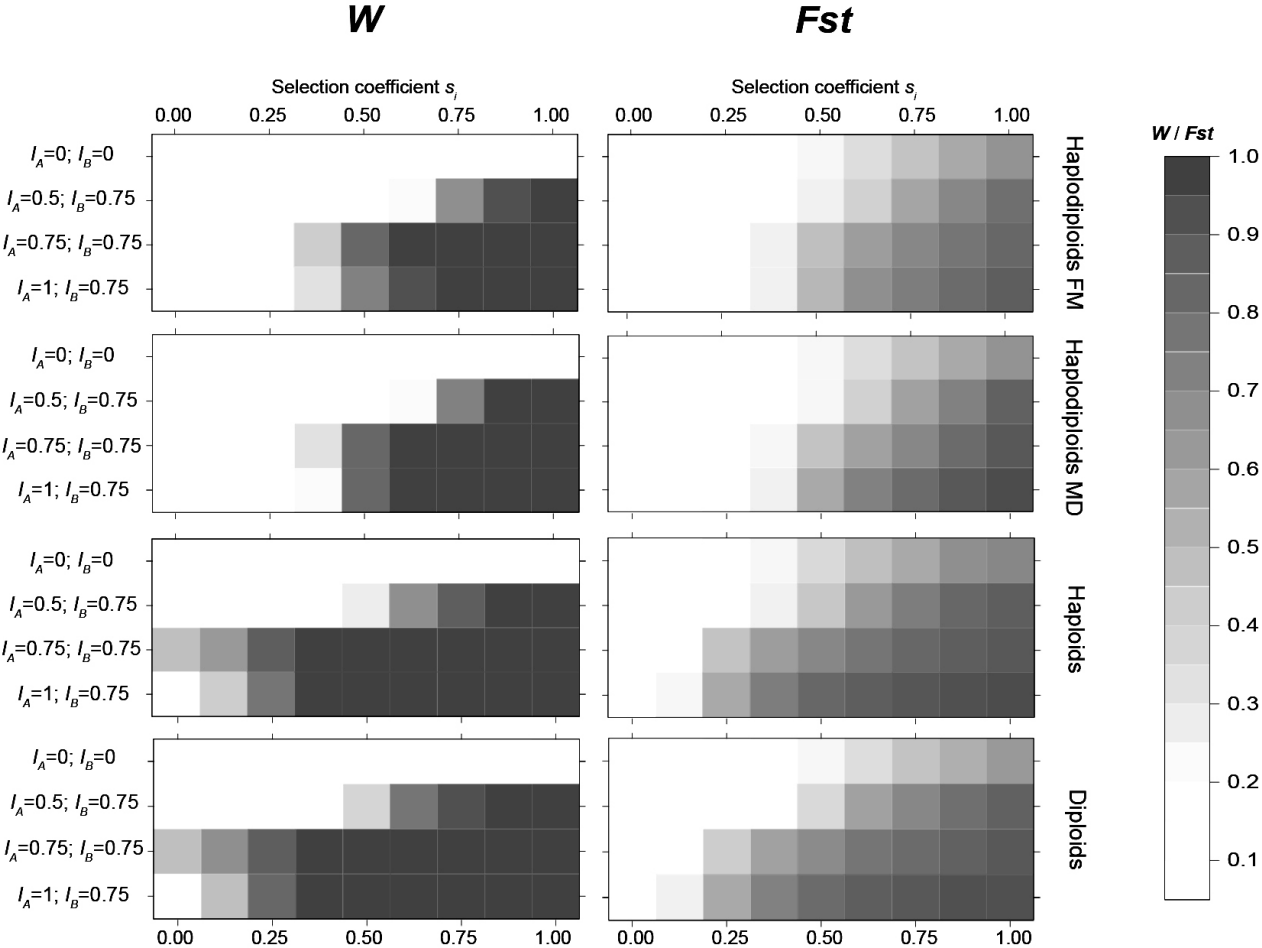
**Effect of the selection coefficient *s*_*i *_on bidirectional CI and genetic divergence**. A representation of the evolution of *W *and *Fst *for different bidirectional CI situations (no CI (*I*_*A *_= *I*_*B *_= 0), (*I*_*A *_= 0.5; *I*_*B *_= 0.75), (*I*_*A *_= 0.75; *I*_*B *_= 0.75) (*I*_*A *_= 1; *I*_*B *_= 0.75)) and selection coefficients *s*_*i*_. Other parameter values are fixed at *m *= 0.1, *P*_*SR *_= 0.75, *χ *= 0, *t *= 1, *g *= 100, *N *= 50. Each graph represents each phenotype tested (Haplodiploids FM and MD, diploids and haploids). *W *is represented on the left and *Fst *on the right. The higher the value for each variable, the darker the box.

Firstly, in qualitative terms, in all genetic systems, *W *increased as the selection coefficient increased (Figure 4). Also, there was more effect of bidirectional CI on *Fst *as the selection coefficient increased.

Secondly, stronger selection was required to reach high values of *W *for haplodiploids. Similarly, the effect of bidirectional CI on *Fst *in haplodiploids occurred for a narrower range of selection coefficient values than for other genetic systems (Table 2).

Thirdly, in diploids and haploids, for *I*_*A *_= 0.75 and *I*_*B *_= 1, enhancement of *Fst *by bidirectional CI was observed without any selection whereas in haplodiploids, it happened only for selection coefficients above 0.25 (Table 2). We also noticed that for *I*_*A *_= 0.5, *I*_*B *_= 0.75 and for *I*_*A *_= 0.75, *I*_*B *_= 0.75, increases in *Fst *were higher for the FM phenotype than for the MD phenotype. In addition, we found that for middle incompatibility rates (*I*_*A *_= 0.5, *I*_*B *_= 0.75) diploids showed a greater increase in genetic divergence than haploids (Table 2). At *s*_*i *_= 0.375 diploids showed a significantly higher *Fst *with CI than without, whereas in haploids it was non significant. In fact, *Fst *was already high for haploids without CI (*Fst *∈ [0.209; 0.225] compared to *Fst *∈ [0.105; 0.115] for diploids) and *Fst *reached almost the same values in diploids and haploids in the case of bidirectional CI (e.g. for *I*_*A *_= 1, *I*_*B *_= 0.75, *Fst *∈ [0.749; 0.770] for haploids and *Fst *∈ [0.763; 0.784] for diploids). Due to the lack of heterozygotes in the haploid system, the purging of disadvantageous alleles was higher and resulted in higher *Fst *values, a phenomenon characteristic of our semi-dominant locus selection model.

The effect of bidirectional CI on differentiation can be summarized as: MD < FM << haploids < diploids.

### Combined effect of sib-mating and bidirectional CI on genetic differentiation (Figure 5)

**Figure 5:**
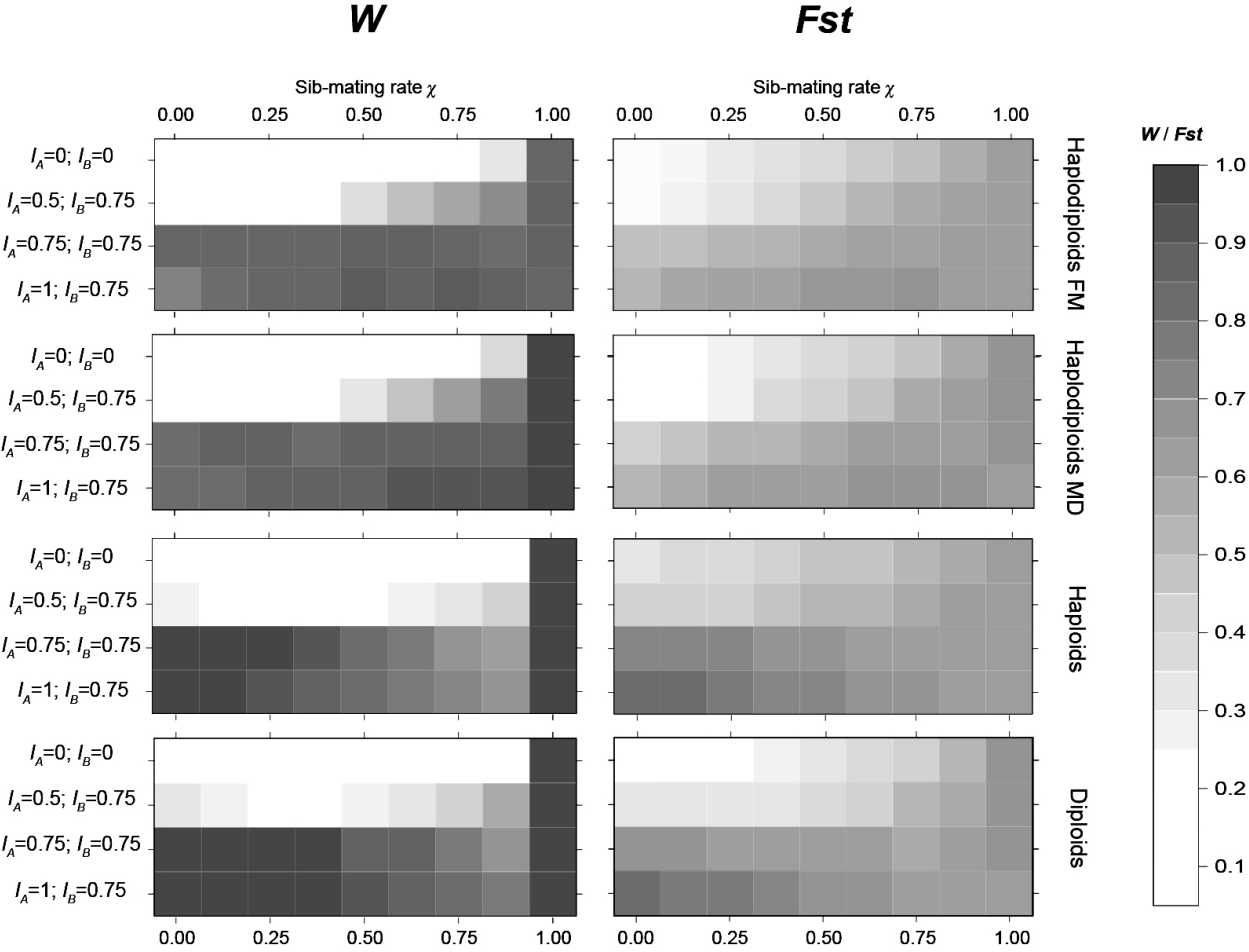
**Effect of the sib-mating rate χ on bidirectional CI and genetic divergence**. A representation of the evolution of *W *and *Fst *for different bidirectional CI situations (no CI (*I*_*A *_= *I*_*B *_= 0), (*I*_*A *_= 0.5; *I*_*B *_= 0.75), (*I*_*A *_= 0.75;*I*_*B *_= 0.75) (*I*_*A *_= 1; *I*_*B *_= 0.75)) and for different values of sib-mating rate *χ*. Other parameter values are fixed at *m *= 0.1, *s*_*i *_= 0.5, *P*_*SR *_= 0.75, *t *= 1, *g *= 100, *N *= 50. Each graph represents each phenotype tested (Haplodiploids FM and MD, diploids and haploids). *W *is represented on the left and *Fst *on the right. The higher the value for each variable, the darker the box.

Firstly, for all values of *χ*, *Fst *and *W *remained higher in the presence of bidirectional CI except for extreme sib-mating situations where they converged. Thus bidirectional CI enhanced genetic differentiation in all genetic systems from low to moderate levels of sib-mating.

Secondly, we observed that interaction between sib-mating and bidirectional CI for *W *values depended on the genetic system being tested (Figure 5). In haplodiploids, sib-mating increased *W *for middle bidirectional CI rate (*I*_*A *_= 0.5; *I*_*B *_= 0.75) but had no effect for higher bidirectional CI rates. In diploids and haploids *W *always showed a minimum at intermediate values of sib-mating. Indeed, it decreased with low levels of sib-mating and increased with high levels of sib-mating. The minimum value of *W *was observed for *χ *= 0.25 for middle bidirectional CI rates (*I*_*A *_= 0.5; *I*_*B *_= 0.75) and for *χ *= 0.875 for higher bidirectional CI rates.

In haplodiploids (Figure 5), sib-mating increased *Fst *while in diploids and haploids, this phenomenon was only observed from middle bidirectional CI (*I*_*A *_= 0.5; *I*_*B *_= 0.75) to no bidirectional CI.

### Effect of transmission efficiency

We also tested the effect of a reduction in transmission efficiency *t *on the different genetic systems. We first observed that *W *decreased for higher values of *t *in haplodiploids compared to the other genetic systems. In the haplodiploid genetic system, maintenance of *Wolbachia *diversity was observed only for values of *t *equal to or above 0.95. For the other genetic systems, maintenance was as low as 0.90. Therefore, the diversity of *Wolbachia *strains can only be maintained in host populations in which transmission efficiency is almost complete, especially in haplodiploid systems.

### Sensitivity of stochastic model to generation length and population size

First, we tested the effect of various population sizes on the results (*N *= 50, 200, 500, 1000). Values of the variables *Fst *and *W *were found to be stable for population size above *N *= 50. We saw a decrease in the standard error of the variables estimation as population size increased because of reduction of genetic drift effect. Variables *W *and *Fst *were found to be slightly lower for population size *N *= 50 but not significantly so.

Second, we increased generation length to see long-term dynamics of the variables *W *and *Fst*. We found that if *W *was close to 1 or equal to 0, it was stable after 100 generations for long time (>2000 generations). For intermediate values recorded at *g *= 100, *W *tended to drop to 0 after a thousand generations. We noted that the observed number of generations before reaching 0 were lower for the MD phenotype than for FM phenotype. We observed also that if we increased population size, we augmented the number of generations necessary for *W *to reach zero. For *Fst*, we observed the same dynamic as *W*, following the decreasing influence of bidirectional CI when *W *decreased.

## Discussion

### Convergence of deterministic and stochastic models

In accordance with previously published models of haploids [28], we first observed that below a threshold migration rate, *Wolbachia *strains were maintained. However our values for the threshold migration rate were lower than the ones observed in the deterministic model of Telschow *et al*. [28] because our approach relies on a stochastic modeling, with a high impact of genetic drift. Therefore it is possible for a *Wolbachia *strain to be lost due to genetic drift if it reaches a low frequency. This was not possible in previous models. We also observed that the threshold migration rates for both the stochastic and previous deterministic approach became closer at higher selection rates. This was due to drift effects on stochastic models that became negligible in comparison to selection at very high values of *s*_*i*_.

We also saw that the *W *variable took a value of 0 or near 1 if the program was run a long duration. This meant that either the *Wolbachia *strain diversity was maintained in almost all repetitions or that one strain invaded both insect populations. It reflects a dynamic where there are two stable equilibria (1) one *Wolbachia *strain in the two populations or (2) two strains present but each strain is restricted to a different population. In this respect, our results are similar to those of other determinist approaches, which have investigated the dynamics of strains harbored by different populations [28,31].

Finally, the only previous model that has considered sib-mating found that *Wolbachia *is harder to spread in populations submitted to sib-mating [39]. It even cannot persist beyond a threshold level of sib-mating because CI expression becomes too rare. An analogous result in our model would have been the invasion of one strain of *Wolbachia *in to both insect populations i.e. *W *= 0. But in fact in our model, selection allows *Wolbachia *to be maintained at very high *χ *values because when the sib-mating rate *χ *is high, *Wolbachia *and the nuclear locus under selection segregate together. However, at near complete sib-mating, it was possible for *Wolbachia *strains to be maintained only if transmission efficiency was total or near-total.

### Specific responses of haplodiploid populations under bidirectional CI

Our model highlights that bidirectional CI in the haplodiploid genetic system is less likely to maintain *Wolbachia *diversity as well as enhance genetic differentiation between differentially infected populations. The possibility of the production of arrhenotokous male following migration in the first generation may explain the lower threshold migration rate within haplodiploid systems. Differentiation in all systems is maintained because first migrant females are more likely to cross with an individual harboring a different *Wolbachia *strain and, hence, more likely to suffer more from CI than a resident female. In haplodiploid genetic systems however, CI produces as many males (FM) or even more males (MD) than in compatible crosses. These males harbor *Wolbachia *from the immigrant population. Therefore, the relative frequency of males harboring immigrant *Wolbachia *is higher in the following generation in haplodiploids than in haploids and diploids. As a result, in the second generation, recently migrated lineages have more available males having the same *Wolbachia *and, therefore, suffer less from CI effect than those of the first generation. These factors facilitate invasion of the immigrant bacteria strain and *Wolbachia *diversity is consequently harder to maintain for haplodiploids.

In parallel, we note that there is a high sensitivity to transmission rate variation in the haplodiploid systems. Within the literature, there are records of transmission rates that exceed 95% (for example 96–97% in *Culex pipiens *[45] or 98.6% in *Drosophila simulans *[46]). Our work has shown that such variations at high rates have a weak impact on *Wolbachia *dynamics in haploid and diploid genetic systems, but a high impact in haplodiploid genetic systems.

As shown in previous models, when *Wolbachia *diversity is maintained, *Fst *increases because bidirectional CI results in migrant offspring death and, hence, in a reduction of the effective migration rate [28,30]. We observed that the effect on genetic divergence was weaker in haplodiploids than in other systems, even when both *Wolbachia *strains were maintained. As for *Wolbachia*, this observation appears to arise from the production of males in incompatible crosses in haplodiploids, allowing more introgression of the immigrant allele to occur.

### Sib-mating compensates for differences between haplodiploids and other systems

Very high values of sib-mating always favor divergence and maintenance of *Wolbachia *infection diversity by maintaining the association between *Wolbachia *and nuclear genes. But at lower values of sib-mating, in haploid and diploid systems, we observed a reduced *W *in the presence of sib-mating. This reduction of *W *due to sib-mating did not occur in haplodiploid systems. However, in haplodiploid systems, sib-mating inhibits the introgression associated with haploid male production. In all systems, when individuals mate with sibs, incompatible crosses occur less frequently and, hence, reduce the impact of bidirectional CI on genetic divergence. But in the case of haplodiploids, as discussed previously, a reduction of *W *in the absence of sib-mating, relies on the production of males from incompatible crosses. These males, in effect, reduce the fitness of local females (i.e. local *Wolbachia*) by causing CI, and, hence favoring the introgression of exotic *Wolbachia *strain. In this situation, sib-mating prevents introgression by affording sibling males a higher reproductive success in comparison to non-sibling males. Thus overall, the effect of sib-mating on *W *is less pronounced in haplodiploids than in haploids or diploids.

### The implications of bidirectional CI on speciation by reinforcement

The present model shows that sib-mating, which is frequently observed in gregarious haplodiploid parasitoids, can complement less efficient bidirectional CI. In this study, we have ascertained that sib-mating is capable of enhancing the weak effect of bidirectional CI in haplodiploid systems. Moreover, in a previous work on unidirectional CI in haplodiploids, sib-mating was shown to reduce the loss of population growth rate via CI during *Wolbachia *invasion [40]. This contribution of sib-mating in the *Wolbachia*-induced differentiation will have an effect on the next step of speciation according to the reinforcement theory. Reinforcement speciation theory predicts that postmating isolation mechanisms, if maintained through generations, will be replaced by less costly premating isolation mechanisms [47]. In the case of postmating isolation induced by bidirectional CI, a previous model has shown that premating isolation mechanisms are likely to be selected for [30]. Nevertheless, evolution of premating isolation (further steps of reinforcement) may be counteracted by sib-mating because sib-mating may limit the cost of *Wolbachia *postmating mechanisms. We therefore expect selection for premating isolation mechanisms to be weaker in haplodiploids encountering frequent sib-mating. Interestingly, in haplodiploid spider mites, it has been demonstrated that *Wolbachia *induces sib-mating in infected individuals [48]. The present model suggests that such induced sib-mating may be an efficient alternative strategy to CI for maintaining *Wolbachia *in a population. In addition, with MD and FM, the other effect of *Wolbachia *in haplodiploids is thelytokous parthenogenesis induction (PI). It can be viewed as the extreme inbreeding strategy and an efficient strategy for *Wolbachia *to maintain strain diversity jointly with local adaptation of its host in parapatric populations. In conclusion, we emphasize that ability for *Wolbachia *to maintain postmating isolation and select for premating isolation mechanisms is dependent on the mating system of the host species. Inbreeding mating systems should be further investigated in future work on *Wolbachia*.

### Reconsidering the *Nasonia *case

The clearest example of speciation likely to have been induced by *Wolbachia *is "the *Nasonia *case". The three species of the *Nasonia *complex, are bidirectionally incompatible, due to either MD or FM [23] and are genetically differentiated [49]. The hypothesis is that bidirectional CI is the major agent responsible for their primary isolation [22,23]. Reinforcement has not occurred totally yet since no premating isolation had arisen. The closest related species, *Nasonia giraulti *and *N. longicornis *occur in allopatry, which may explain the absence of premating isolation. However, these two species can occur in sympatry with the more distantly related species *N. vitripennis *but no premating isolation mechanisms have evolved. We can speculate in light of our results as to what the predominant role of bidirectional CI *versus *the important contribution of sib-mating in *Nasonia *speciation is by considering that: 

– in haplodiploids, *Wolbachia *induced bidirectional CI alone is less efficient to contribute to genetic divergence.

-*Nasonia *species are gregarious and exhibit sib-mating behavior, and even within host mating, when females emerged already mated from the host puparium [50].

Our hypothesis is that bidirectional CI and sib-mating have in combination contributed to genetic divergence of *Nasonia *species and that this has occurred in a framework in which frequent sib-mating has prevented evolution of premating isolation mechanisms.

### Implications for biological control strategies using parasitoid agents

CI inducing *Wolbachia *have already been used as a biological control strategy to control insect pest growth rate by introducing an infected conspecific [51,52]. Biological control programs that utilize endemic wasp species can conceivably make use of the knowledge that *Wolbachia *could play a role in local adaptation. Introduced biological control agents often interact with local populations that are specialized on a different host, or can be composed of different genotypes reflecting virulence on different hosts [53,54]. Given that sib-mating coupled with bidirectional CI contribute to maintaining host specialization, it may be envisaged to introduce agents with different host niche and infected with different *Wolbachia *strains. If bacteria strains are bidirectionally incompatible, then reproductive isolation will occur. On the one hand, too many incompatible crosses can render bidirectional CI deleterious due to the reduction in population growth associated with it. On the other hand, a sib-mating process can compensate for these same detrimental effects. Local adaptation is, therefore, reinforced over generations so that mixing with locally avirulent populations is avoided. Existing biological control strategies may offer the opportunity to field-test the scenarios developed in this model.

## Conclusion

Our results show that the implications of *Wolbachia *in genetic differentiation depend on crucial interaction between biological traits such as the genetic system and sib-mating rate. Complex analysis of these factors reveals a general trend that permits two principal conclusions to be drawn. Firstly, in haplodiploid systems bidirectional CI possesses a limited ability to maintain *Wolbachia *diversity and, by extrapolation, a limited ability to maintain genetic divergence of locally adapted genes. Secondly, sib-mating behavior is particularly efficient in haplodiploid systems to counteract a reduced impact of bidirectional CI on genetic divergence. However, it may slow down the speciation process by reducing the postmating isolation cost of *Wolbachia *and, thereby, the selection for premating isolation. Therefore, speciation induced by bidirectional CI alone is expected to occur less frequently and for a narrower range of parameters in haplodiploids compared to diploids.

## Authors' contributions

All the authors, AB, SD, FV and JFS, participated in the design of the study and drafted the article. In addition, AB wrote the R source code with the *CIParasitoid *package and analyzed the output data. SD also contributed to the development of the R source code.

## Supplementary Material

Additional file 1 **R package *CIParasitoid *for Windows XP**. Package *CIParasitoid *for R containing the program presented here. It has been built on R 2.8.0 for Windows XP. The latest version of R along with installation instructions can be found at . Click here for file

Additional file 2 **R package *CIParasitoid *for Mac OS X Intel processor**. Package *CIParasitoid *for R containing the program presented here. It has been built on R 2.8.0 for MacOSX Intel processor. The latest version of R along with installation instructions can be found at . First extract the tar.gz binary file from the .zip file before any installation in R. Click here for file

Additional file 3 **R package *CIParasitoid *for Linux**. Package *CIParasitoid *for R containing the program presented here. It has been built on R 2.8.0 for Linux. The latest version of R along with installation instructions can be found at . First extract the tar.gz binary file from the .zip file before any installation in R. Click here for file

Additional file 4 **R package *CIParasitoid *for Mac OS X PowerPC processor**. Package *CIParasitoid *for R containing the program presented here. It has been built on R 2.8.0 for MacOSX PowerPC processor. The latest version of R along with installation instructions can be found at . First extract the tar.gz binary file from the .zip file before any installation in R. Click here for file
