# Supplementary material for: Maintenance of adaptive differentiation by Wolbachia induced bidirectional cytoplasmic incompatibility: the importance of sib-mating and genetic systems
Source: BMC Evol Biol. 2009 Aug 4;9:185. doi: 10.1186/1471-2148-9-185 (PMC2738673; doi:10.1186/1471-2148-9-185)
Supplement: Additional file 1 — R package CIParasitoid for Windows XP. Package CIParasitoid for R containing the program presented here. It has been built on R 2.8.0 for Windows XP. The latest version of R along with installation instructions can be found at . [file 1471-2148-9-185-S1.zip › CIParasitoid/html/00Index.html]

R: Simulation of bidirectional Cytoplasmic Incompatibility in
parasitoid population

# Simulation of bidirectional Cytoplasmic Incompatibility in parasitoid population

---

## Documentation for package ‘CIParasitoid’ version 1.0

## Help Pages

|  |  |
| --- | --- |
| CIParasitoid | Simulations of Unidirectional and Bidirectional Cytoplasmic Incompatibility in parasitoid populations |
| CIParasitoidDiplo | Stochastic simulation of diploid populations under bidirectional CI |
| CIParasitoidFemMor | Stochastic simulation of haplodiploid populations submitted to bidirectional CI-Female Mortality Phenotype |
| CIParasitoidHaplo | Stochastic simulation of haploid populations under bidirectional CI |
| CIParasitoidMalDev | Stochastic simulation of a haplodiploid populations submitted to bidirectional CI-Male Development Phenotype |
| FathSampleP | Sampling of males participating in reproduction (parapatry version) |
| frqCrV1P | Calculation of virulence alleles frequencies (parapatry version) |
| frqWbP | Calculation of Wolbachia frequencies (parapatry version) |
| MigrSample | Sample of migrant population |
| MothSampleHaplo | Sampling of females participating in reproduction for haploid model |
| MothSampleP | Sampling of females participating in reproduction (parapatry version) |
| NbSexIndP | Recording position of individuals (parapatry version) |
| reproP | Descendant genotypes sample (parapatry version) |
| sex | Sample sex of individuals |
