# Supplementary material for: Maintenance of adaptive differentiation by Wolbachia induced bidirectional cytoplasmic incompatibility: the importance of sib-mating and genetic systems
Source: BMC Evol Biol. 2009 Aug 4;9:185. doi: 10.1186/1471-2148-9-185 (PMC2738673; doi:10.1186/1471-2148-9-185)
Supplement: Additional file 1 — R package CIParasitoid for Windows XP. Package CIParasitoid for R containing the program presented here. It has been built on R 2.8.0 for Windows XP. The latest version of R along with installation instructions can be found at . [file 1471-2148-9-185-S1.zip › CIParasitoid/html/CIParasitoidFemMor.html]

R: Stochastic simulation of haplodiploid populations submitted to bidirectional CI-Female Mortality Phenotype

|  |  |
| --- | --- |
| CIParasitoidFemMor {CIParasitoid} | R Documentation |

## Stochastic simulation of haplodiploid populations submitted to bidirectional CI-Female Mortality Phenotype

### Description

Main function for the simulation of two haplodiploid populations where Cytoplasmic Incompatibility occur. Each population is adapted to its environment on a virulence locus (selection on female). Populations are exchanging genes through migration. Bidirectional CI phenotype is Female Mortality. It means that diploid eggs (females) die and males develop in incompatible crosses.

### Usage

```
CIParasitoidFemMor(Gmax,nrep,popsize,m,tinfest,chi,Psr,I,s,output,t,PLOT,path)
```

### Arguments

|  |  |
| --- | --- |
| `Gmax` | an integer corresponding to the number of generations. |
| `nrep` | an integer corresponding to the total number of repetitions. |
| `popsize` | an integer corresponding to the number of individuals per population. |
| `m` | a numeric corresponding to migration rate between population. |
| `tinfest` | a numeric corresponding to the rate of infestation at starting generation i.e. proportion of Wolbachia in each population. |
| `chi` | a numeric corresponding to the rate of sib-mating crosses. |
| `Psr` | a numeric corresponding to sexratio (proportion of female). |
| `I` | a vector of length 2 corresponding to cytoplasmic incompatibility rate.nth value correspond to value for nth strain of bacteria. |
| `s` | a vector of length 2 corresponding to strength of selection in populations.nth value correspond to value for nth population. |
| `output` | a character chain corresponding to file name for output. |
| `t` | a numeric corresponding to transmission efficiency of Wolbachia. |
| `PLOT` | a logical, TRUE if plot are desired during simulation and FALSE otherwise. Plots are the evolution of Virulent allele and A Wolbachia strain in the two populations. |
| `path` | a character chain corresponding to path for outputs. |

### Value

The file `output` contains values:

|  |  |
| --- | --- |
| `nrep` | Number of repetition |
| `IA` | Cytoplasmic Incompatibility of strain 1 |
| `IB` | Cytoplasmic Incompatibility of strain 2 |
| `VirPop1` | Frequency of virulent allele in population 1 |
| `VirPop2` | Frequency of virulent allele in population 2 |
| `WbAPop1` | Frequency of Wolbachia strain 1 in population 1 |
| `WbAPop2` | Frequency of Wolbachia strain 1 in population 2 |
| `AvirPop1` | Frequency of avirulent allele in population 1 |
| `AvirPop2` | Frequency of avirulent allele in population 2 |
| `WbBPop1` | Frequency of Wolbachia strain 2 in population 1 |
| `WbBPop2` | Frequency of Wolbachia strain 2 in population 2 |

### Author(s)

Antoine Branca

### References

Branca A., Vavre F., Silvain J.-F., Dupas S., Maintenance of adaptive differentiation by Wolbachia induced bidirectional cytoplasmic incompatibility: sib-mating and reproductive system matter, 2008, submitted

### See Also

`frqWbP`,`frqCrV1P`,`reproP` ,`NbSexIndP`,`sex`,`MothSampleP`,`FathSampleP`,`MigrSample`

### Examples

```
CIParasitoidFemMor(Gmax=10,nrep=2,popsize=50,m=0.1,tinfest=1,chi=0,Psr=0.4,I=c(0.8,0.8),s=c(0.1,0.1),output="testMD.txt",t=1,PLOT=TRUE,path=getwd())
```

---

[Package *CIParasitoid* version 1.0 Index]
