# Supplementary material for: Maintenance of adaptive differentiation by Wolbachia induced bidirectional cytoplasmic incompatibility: the importance of sib-mating and genetic systems
Source: BMC Evol Biol. 2009 Aug 4;9:185. doi: 10.1186/1471-2148-9-185 (PMC2738673; doi:10.1186/1471-2148-9-185)
Supplement: Additional file 1 — R package CIParasitoid for Windows XP. Package CIParasitoid for R containing the program presented here. It has been built on R 2.8.0 for Windows XP. The latest version of R along with installation instructions can be found at . [file 1471-2148-9-185-S1.zip › CIParasitoid/html/FathSampleP.html]

R: Sampling of males participating in reproduction (parapatry version)

|  |  |
| --- | --- |
| FathSampleP {CIParasitoid} | R Documentation |

## Sampling of males participating in reproduction (parapatry version)

### Description

Random sampling of the males participating in reproduction in local population.Migrant males are sample according to migration rate m.
It is called through `CIParasitoidDiplo`, `CIParasitoidFemMor`, `CIParasitoidHaplo`, `CIParasitoidMalDev`.

### Usage

```
FathSampleP(popnumber,popsize,m,nummals)
```

### Arguments

|  |  |
| --- | --- |
| `popnumber` | an integer corresponding to the total number of population. |
| `popsize` | an integer corresponding to the size of population. |
| `m` | a numeric corresponding to migration rate. |
| `nummals` | a vector containing positions of males in matrix of sexe, virulence genotype and Wolbachia status. |

### Value

Return two vectors:

|  |  |
| --- | --- |
| `ppere` | contains number of population of sample males. |
| `npere` | contains position of sample males in `ppere` population. |

### Warning

Return multi-arguments that provokes a warning message at each loop

### Note

section{Warning }{Return multi-arguments that provokes a warning message at each loop}

### Author(s)

Antoine Branca

### See Also

`MothSampleP`

---

[Package *CIParasitoid* version 1.0 Index]
