# Supplementary material for: Maintenance of adaptive differentiation by Wolbachia induced bidirectional cytoplasmic incompatibility: the importance of sib-mating and genetic systems
Source: BMC Evol Biol. 2009 Aug 4;9:185. doi: 10.1186/1471-2148-9-185 (PMC2738673; doi:10.1186/1471-2148-9-185)
Supplement: Additional file 1 — R package CIParasitoid for Windows XP. Package CIParasitoid for R containing the program presented here. It has been built on R 2.8.0 for Windows XP. The latest version of R along with installation instructions can be found at . [file 1471-2148-9-185-S1.zip › CIParasitoid/html/frqCrV1P.html]

R: Calculation of virulence alleles frequencies (parapatry version)

|  |  |
| --- | --- |
| frqCrV1P {CIParasitoid} | R Documentation |

## Calculation of virulence alleles frequencies (parapatry version)

### Description

Calculate frequencies of virulence alleles inside a population.
It is called through `CIParasitoidDiplo`, `CIParasitoidFemMor`, `CIParasitoidHaplo`, `CIParasitoidMalDev`.

### Usage

```
frqCrV1P(AllVir, popsize)
```

### Arguments

|  |  |
| --- | --- |
| `AllVir` | a vector of integer containing allele on virulence locus for each individual.Values are: -1 for allele selected in population 1 -2 for allele selected in population 2 -0 for second allele of males (males are haploid) |
| `popsize` | an integer corresponding to the size of population. |

### Value

A vector of length 2:

|  |  |
| --- | --- |
| -value 1 | is frequency of allele selected in population 1; |
| -value 2 | is frequency of allele selected in population 2. |

### Author(s)

Antoine Branca

### See Also

See Also `frqWbP`, `CIParasitoidDiplo`, `CIParasitoidFemMor`, `CIParasitoidHaplo`, `CIParasitoidMalDev`

---

[Package *CIParasitoid* version 1.0 Index]
