# Supplementary material for: Maintenance of adaptive differentiation by Wolbachia induced bidirectional cytoplasmic incompatibility: the importance of sib-mating and genetic systems
Source: BMC Evol Biol. 2009 Aug 4;9:185. doi: 10.1186/1471-2148-9-185 (PMC2738673; doi:10.1186/1471-2148-9-185)
Supplement: Additional file 1 — R package CIParasitoid for Windows XP. Package CIParasitoid for R containing the program presented here. It has been built on R 2.8.0 for Windows XP. The latest version of R along with installation instructions can be found at . [file 1471-2148-9-185-S1.zip › CIParasitoid/html/frqWbP.html]

R: Calculation of Wolbachia frequencies (parapatry version)

|  |  |
| --- | --- |
| frqWbP {CIParasitoid} | R Documentation |

## Calculation of Wolbachia frequencies (parapatry version)

### Description

Calculate frequencies of Wolbachia inside a population. It is called through `CIParasitoidDiplo`, `CIParasitoidFemMor`, `CIParasitoidHaplo`, `CIParasitoidMalDev`.

### Usage

```
frqWbP(wolb, popsize)
```

### Arguments

|  |  |
| --- | --- |
| `wolb` | a vector of integer containing Wolbachia infection for each individual. Values are 1 for A strain infection, 2 for B strain infection, 3 for no infection. |
| `popsize` | an integer corresponding to the size of population. |

### Value

A vector of length 3:

|  |  |
| --- | --- |
| -value 1 | is A strain frequency; |
| -value 2 | is B strain frequency; |
| -value 3 | is Wolbachia-free individuals frequency. |

### Author(s)

Antoine Branca

### References

Not yet...

### See Also

See Also `frqCrV1P`, `CIParasitoidDiplo`, `CIParasitoidFemMor`, `CIParasitoidHaplo`, `CIParasitoidMalDev`

---

[Package *CIParasitoid* version 1.0 Index]
