# Supplementary material for: Maintenance of adaptive differentiation by Wolbachia induced bidirectional cytoplasmic incompatibility: the importance of sib-mating and genetic systems
Source: BMC Evol Biol. 2009 Aug 4;9:185. doi: 10.1186/1471-2148-9-185 (PMC2738673; doi:10.1186/1471-2148-9-185)
Supplement: Additional file 1 — R package CIParasitoid for Windows XP. Package CIParasitoid for R containing the program presented here. It has been built on R 2.8.0 for Windows XP. The latest version of R along with installation instructions can be found at . [file 1471-2148-9-185-S1.zip › CIParasitoid/html/MothSampleHaplo.html]

R: Sampling of females participating in reproduction for haploid model

|  |  |
| --- | --- |
| MothSampleHaplo {CIParasitoid} | R Documentation |

## Sampling of females participating in reproduction for haploid model

### Description

Random sampling of the females participating in reproduction in local population.Migrant females are sample according to migration rate m.
It is call through `CIParasitoidHaplo`.

### Usage

```
MothSampleHaplo(popnumber,popsize,m,numfems,AllVir,selec,fitness)
```

### Arguments

|  |  |
| --- | --- |
| `popnumber` | an integer corresponding to the total number of population. |
| `popsize` | an integer corresponding to the size of population. |
| `m` | a numeric corresponding to migration rate. |
| `numfems` | a vector containing positions of females in matrix of sexe, virulence genotype and Wolbachia status. |
| `AllVir` | a vector of integer containing allele on virulence locus for each individual.Values are 1 for allele selected in pop 1, 2 for allele selected in pop 2 and 0 for second allele of males (males are haploid). |
| `selec` | a vector of size popnumber corresponding to direction of selection:-value 1 correspond to selection toward virulence (allele 1) -value 2 correspond to selection toward avirulence (allele 2) |
| `fitness` | a vector of size popnumber corresponding to strength of selection: females have a probability of (1-fitness)/(total number of females) to be chosen for reproduction in unadapted population. |

### Value

|  |  |
| --- | --- |
| `pmere` | contains number of population of sample females. |
| `nbmere` | contains position of sample females in `pmere` population. |

### Warning

Return multi-arguments that provokes a warning message at each loop

### Note

section{Warning }{Return multi-arguments that provokes a warning message at each loop}

### Author(s)

Antoine Branca

### See Also

`FathSampleP`

---

[Package *CIParasitoid* version 1.0 Index]
