# Supplementary material for: Maintenance of adaptive differentiation by Wolbachia induced bidirectional cytoplasmic incompatibility: the importance of sib-mating and genetic systems
Source: BMC Evol Biol. 2009 Aug 4;9:185. doi: 10.1186/1471-2148-9-185 (PMC2738673; doi:10.1186/1471-2148-9-185)
Supplement: Additional file 1 — R package CIParasitoid for Windows XP. Package CIParasitoid for R containing the program presented here. It has been built on R 2.8.0 for Windows XP. The latest version of R along with installation instructions can be found at . [file 1471-2148-9-185-S1.zip › CIParasitoid/html/reproP.html]

R: Descendant genotypes sample (parapatry version)

|  |  |
| --- | --- |
| reproP {CIParasitoid} | R Documentation |

## Descendant genotypes sample (parapatry version)

### Description

This function fills genotype table for each generation according to parental genotypes. It is called through `CIParasitoidDiplo`, `CIParasitoidFemMor`, `CIParasitoidHaplo`, `CIParasitoidMalDev`.

### Usage

```
reproP(tab, nbm, nbp, pp, pm, sxtemp)
```

### Arguments

|  |  |
| --- | --- |
| `tab` | a list containing:- K1 vector genotype on chromosome 1;- K2 vector genotype on chromosome 2. |
| `tabgm` | a list containing genotype of great mother:- K1 vector genotype on chromosome 1- K2 vector genotype on chromosome 2. |
| `nbm` | an integer corresponding to the number of the female. |
| `nbp` | an integer corresponding to the number of the male. |
| `sxtemp` | an integer corresponding to the sex of individual:- 0 for a male- for a female |
| `sib` | a logical TRUE if the cross is between sibling and FALSE of not. |

### Value

A vector of numeric of length 2 corresponding to genotype:

|  |  |
| --- | --- |
| -value 1 | is for chromosome 1; |
| -value 2 | is for chromosome 2. |

### Author(s)

Antoine Branca

---

[Package *CIParasitoid* version 1.0 Index]
