# Supplementary material for: Maintenance of adaptive differentiation by Wolbachia induced bidirectional cytoplasmic incompatibility: the importance of sib-mating and genetic systems
Source: BMC Evol Biol. 2009 Aug 4;9:185. doi: 10.1186/1471-2148-9-185 (PMC2738673; doi:10.1186/1471-2148-9-185)
Supplement: Additional file 2 — R package CIParasitoid for Mac OS X Intel processor. Package CIParasitoid for R containing the program presented here. It has been built on R 2.8.0 for MacOSX Intel processor. The latest version of R along with installation instructions can be found at . First extract the tar.gz binary file from the .zip file before any installation in R. [file 1471-2148-9-185-S2.zip › CIParasitoid_1.0_MacIntel.tar.gz!/CIParasitoid/html/CIParasitoid-package.html]

R: Simulations of Unidirectional and Bidirectional Cytoplasmic Incompatibility in parasitoid populations

|  |  |
| --- | --- |
| CIParasitoid-package {CIParasitoid} | R Documentation |

## Simulations of Unidirectional and Bidirectional Cytoplasmic Incompatibility in parasitoid populations

### Description

This package provides a tool for simulate populations of parasitoids submitted to Cytoplasmic Incompatibility. All possible ploidy levels have been implemented: diploid, haploid and haplodiploid. Simulations start after that two populations have evolved separately and come in secondary contact. Each individual is then adapted to different environment at a single virulence locus (i.e. capacity for successfully develop its progeny in the host). During allopatric divergence, each population acquired a different Wolbachia strain potentially bidirectionally incompatible.

### Details

Main functions of this package are `CIParasitoidDiplo` for diploids, `CIParasitoidHaplo` for haploids, `CIParasitoidFemMor` for Female Mortality phenotype in haplodiploids, `CIParasitoidMalDev` for Male Development phenotype in haplodiploids.

### Author(s)

Antoine Branca

### References

Branca A., Vavre F., Silvain J.-F., Dupas S. 2008 Maintenance of adaptive differentiation by Wolbachia induced bidirectional cytoplasmic incompatibility: sib-mating and reproductive system matter

### See Also

`CIParasitoidDiplo`, `CIParasitoidFemMor`, `CIParasitoidHaplo`, `CIParasitoidMalDev`

### Examples

```
#This values show a short-in-time example of each function
 
CIParasitoidDiplo(Gmax=10,nrep=2,popsize=50,m=0.1,tinfest=1,chi=0,Psr=0.4,I=c(0.8,0.8),s=c(0.1,0.1),output="testDip.txt",t=1,PLOT=TRUE,path=getwd())
CIParasitoidFemMor(Gmax=10,nrep=2,popsize=50,m=0.1,tinfest=1,chi=0,Psr=0.4,I=c(0.8,0.8),s=c(0.1,0.1),output="testFM.txt",t=1,PLOT=TRUE,path=getwd())
CIParasitoidMalDev(Gmax=10,nrep=2,popsize=50,m=0.1,tinfest=1,chi=0,Psr=0.4,I=c(0.8,0.8),s=c(0.1,0.1),output="testMD.txt",t=1,PLOT=TRUE,path=getwd())
CIParasitoidHaplo(Gmax=10,nrep=2,popsize=50,m=0.1,tinfest=1,chi=0,Psr=0.4,I=c(0.8,0.8),s=c(0.1,0.1),output="testHap.txt",t=1,PLOT=TRUE,path=getwd())
```

---

[Package *CIParasitoid* version 1.0 Index]
