# Supplementary material for: Maintenance of adaptive differentiation by Wolbachia induced bidirectional cytoplasmic incompatibility: the importance of sib-mating and genetic systems
Source: BMC Evol Biol. 2009 Aug 4;9:185. doi: 10.1186/1471-2148-9-185 (PMC2738673; doi:10.1186/1471-2148-9-185)
Supplement: Additional file 2 — R package CIParasitoid for Mac OS X Intel processor. Package CIParasitoid for R containing the program presented here. It has been built on R 2.8.0 for MacOSX Intel processor. The latest version of R along with installation instructions can be found at . First extract the tar.gz binary file from the .zip file before any installation in R. [file 1471-2148-9-185-S2.zip › CIParasitoid_1.0_MacIntel.tar.gz!/CIParasitoid/html/sex.html]

R: Sample sex of individuals

|  |  |
| --- | --- |
| sex {CIParasitoid} | R Documentation |

## Sample sex of individuals

### Description

Sample sex of individuals according to sexratio and val.It is called through `CIParasitoidDiplo`, `CIParasitoidFemMor`, `CIParasitoidHaplo`, `CIParasitoidMalDev`.

### Usage

```
sex(val, sx)
```

### Arguments

|  |  |
| --- | --- |
| `val` | an integer: if val>0 then sexe is male else it is randomly chosen. |
| `sx` | a numeric corresponding to sexratio. |

### Value

Return 0 for a male and 1 for a female.

### Author(s)

Antoine Branca

---

[Package *CIParasitoid* version 1.0 Index]
